# Enabling Covert Body Area Network using Electro-Quasistatic Human Body Communication

**DOI:** 10.1038/s41598-018-38303-x

**Published:** 2019-03-11

**Authors:** Debayan Das, Shovan Maity, Baibhab Chatterjee, Shreyas Sen

**Affiliations:** Purdue University, School of Electrical and Computer Engineering, West Lafayette, IN 47907 USA

**Keywords:** Biomedical engineering, Applied physics, Biological physics

## Abstract

Radiative communication using electro-magnetic (EM) fields amongst the wearable and implantable devices act as the backbone for information exchange around a human body, thereby enabling prime applications in the fields of connected healthcare, electroceuticals, neuroscience, augmented and virtual reality. However, owing to such radiative nature of the traditional wireless communication, EM signals propagate in all directions, inadvertently allowing an eavesdropper to intercept the information. In this context, the human body, primarily due to its high water content, has emerged as a medium for low-loss transmission, termed human body communication (HBC), enabling energy-efficient means for wearable communication. However, conventional HBC implementations suffer from significant radiation which also compromises security. In this article, we present Electro-Quasistatic Human Body Communication (EQS-HBC), a method for localizing signals within the body using low-frequency carrier-less (broadband) transmission, thereby making it extremely difficult for a nearby eavesdropper to intercept critical private data, thus producing a covert communication channel, i.e. the human body. This work, for the first time, demonstrates and analyzes the improvement in private space enabled by EQS-HBC. Detailed experiments, supported by theoretical modeling and analysis, reveal that the quasi-static (QS) leakage due to the on-body EQS-HBC transmitter-human body interface is detectable up to <0.15 *m*, whereas the human body alone leaks only up to ~0.01 m, compared to >5 *m* detection range for on-body EM wireless communication, highlighting the underlying advantage of EQS-HBC to enable covert communication.

## Introduction

Future advancements of societally critical applications such as connected healthcare, electroceuticals, neuroscience, augmented and virtual reality rely on small form-factor wearables^1,2^, implantables, injectables, ingestibles, and other on-body internet-connected devices, triggering the need for energy-efficient and secure mechanisms for information exchange^3^. This trend has transformed the human body to an integrated network of electronic devices that includes biomedical sensors, closed-loop neuromodulation systems, smartwatches, glasses, or even mobile phones. Wireless Body Area Network (WBAN) has been the de-facto standard for connecting these tiny energy-sparse devices. However, wireless communication using EM fields is fundamentally radiative and attenuates in power density as it propagates through space. Due to this radiative nature of WBAN, the signals require high transmission power and they propagate in all directions. These EM emanations can be easily intercepted by any malicious eavesdropper, who wants to gain access to the critical private information. Hence, data encryption becomes necessary in case of wireless communication. However, such information-theoretic secrecy do not mitigate the threat to user’s privacy^4^ from the very existence of the message itself. Moreover, even the most theoretically robust cryptographic algorithm can often be defeated by an adversary using non-computational techniques such as side-channel analysis.

Quite recently, bio-physical communication using the human body has gained prominence as an energy-efficient information exchange modality^5,6^, as the high-water content of our body provides a low-loss channel for signal propagation. Human Body Communication was first proposed as a method to connect devices on a Personal Area Network (PAN) by Zimmerman *et al*.^7^. Both the transmitter and the receiver are electrically isolated and battery powered devices. The transmitter capacitively couples a narrowband signal on the surface of the human body creating electric fields and the displacement current is picked up at the receiver^8,9^. The closed loop path is formed by the capacitive coupling between earth’s ground and ground electrode of the devices^10,11^. Due to the capacitive return path, this mode is often referred to as capacitive HBC. Oberle^12^ and Wegmueller *et al*.^13^ investigated galvanic coupling in which the signal is applied between two electrodes of the transmitter in direct contact with the human body, and the potential difference generated by the induced electric fields from signal source is sensed by the receiver electrodes on other side of the body. Such differential excitation and termination mode is commonly referred to as galvanic HBC. In the case of galvanic coupling, most of the current flows between the two electrodes of the transmitter device, as it forms a low resistance path for the induced electric current. This increases the loss in galvanic coupling as the distance between the transmitter and receiver increases, making galvanic HBC unsuitable for long-distance on-body communication from a small transmit device. Based on these two types of HBC, there have been several studies characterizing the channel loss and signal transmission mechanisms for intra-body HBC^14,15^. Analysis of EM wave interactions with the human body has also been extensively studied^16–19^.

However, most previous efforts have been focused on narrowband human body communication (NB-HBC). NB-HBC couples modulated narrowband EM signals (20–80 MHz carrier frequency) to the human body using a coupler instead of radiating it with an antenna. The energy consumption of traditional WBANs is in the order of ~$10\,nJ/bit$^20^, whereas NB-HBC techniques consume ~$110\,pJ/bit$^21^. We have recently demonstrated that broadband HBC could reduce this energy-efficiency to <$10\,pJ/bit$^22^. It implies that for a mm^3^-sized battery with 2*J* of available energy, WBAN can only support ~2 *s* of data transfer at a data rate of 100 *Mbps*, whereas a HBC enabled device can support up to ~2000 *s* for the same data rate. Although NB-HBC is more energy-efficient than WBAN, the EM nature of communication radiates significant amount of signal outside the body, thus not maintaining the data privacy.

**Figure 1:**
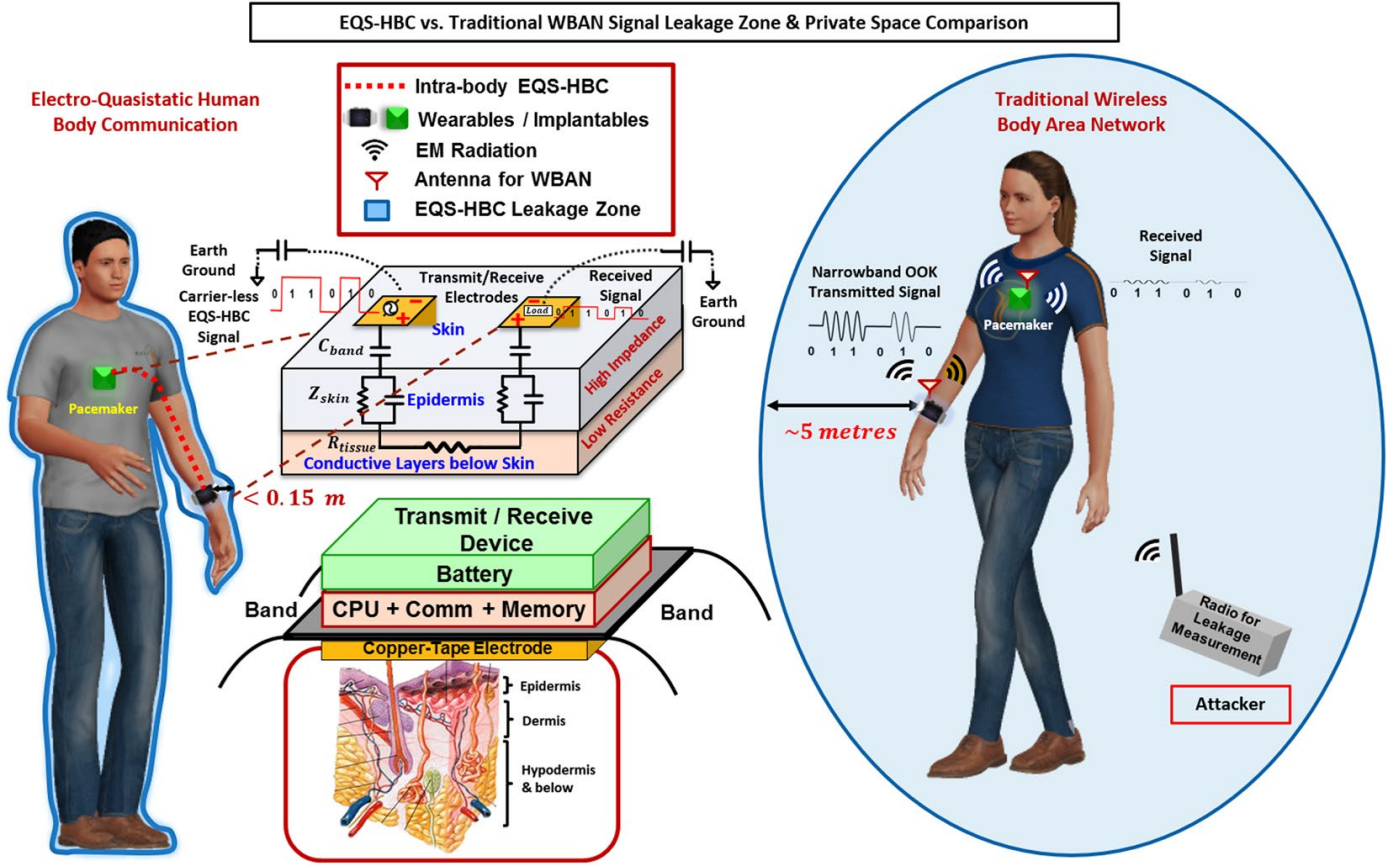
EQS-HBC vs. WBAN: An Overview of the Data Privacy Space. Persons wearing transmitter device (pacemaker) and an on-body hub communicating using EQS-HBC (left) and WBAN (right) respectively. For the intra-body EQS-HBC, signals are coupled to the surface of the human body using an interfacing copper electrode which protrudes from beneath the transmitter consisting of communication module, processing module, memory, and power source. The transmitted signal flows through the low resistance layers of the body below the skin and is picked up by the receiver electrode. On the other hand, WBAN uses an antenna to radiate the signals wirelessly up to a larger distance that can be picked up by a nearby eavesdropper. The privacy space in case of EQS-HBC (<0.15 *m*) is significantly improved by an order of >30×, compared to WBAN (~5 *m*). *The human figures were created using the open*-*source software* ‘MakeHuman’^42^. *The detailed anatomy of the human skin layer structure can be found in*^43^.

In this work, we present a private body area network utilizing Electro-Quasistatic (EQS) transmission to enable physical-layer covert and secure communication. EQS-HBC uses electro-quasistatic signal transmission through the conductive layers below skin, and capacitive return paths with carrier-less signals at frequencies below 1 *MHz*. As shown in Fig. 1, EQS-HBC couples the carrier-less signals through skin layer to the conductive layers below the skin. The coupled signals create an electro-quasistatic (EQS) field throughout the body and the potential difference thus created can be picked up by wearables and implantables distributed around the body. Privacy is essentially enabled by a combination of electro-quasistatic nature of signals involved, ensuring communication signals are not radiated out, along with signal transmission through the conductive layers below the skin, which ensures critical signals stay mostly within the body. Here we use EQS-HBC to reduce signal leakage, making the physical signals hide under ambient noise from a nearby attacker’s perspective, enabling covert communication.

We observe that carrier-less EQS-HBC data transfer maximizes the information capacity of the channel compared to the narrowband signaling (NB-HBC), as it utilizes the full bandwidth instead of a fractional-bandwidth around a carrier frequency. Correspondingly, to achieve the same data rate, wireless signals as well as NB- HBC need to choose a high enough carrier frequency (*f*_*c*_) so that the fractional BW at that frequency equals the EQS human body communication capacity. The high *f*_*c*_ leads to higher EM leakage and hence NB-HBC lacks the inherent physical security.

Data security and privacy is a critical aspect for most human-centered applications including wearable medical devices used for patient monitoring^23,24^, e.g. a doctor reprogramming a pacemaker using the patient’s smartwatch. If the wireless data transmissions can be confined within the human body, it would enable a form of physical-layer covert and inherently secure communication that is currently non-existent on wearable and implantable devices. EQS-HBC presents itself as a strong candidate for enabling covert communication and this work explores the security and privacy aspects of EQS-HBC. Although the magnetic fields are very weak due to the electro-quasistatic nature of transmission, an external eavesdropper can try to detect the quasi-static (QS) leakage (QSL). This work presents various experiments in both time and frequency domains using custom-designed EQS-HBC device, supported by theoretical leakage models to analyze this side-channel QS leakage, and demonstrates the privacy and security of human body data transmission using EQS-HBC.

## Electro-Quasistatic Human Body Communication (EQS-HBC): Fundamentals

EQS-HBC uses electro-quasistatic transmission through the conductive layers below skin, and capacitive return paths with carrier-less signals at frequencies below 1 *MHz*.

### Signal transmission through Conductive layers below Skin

In this work, the signals are capacitively coupled to the epidermal skin layers of the human body which then flows through the conductive layers below of the skin and is finally picked up capacitively at different on-body receivers. The magnitude of the skin impedance (*Z*_*skin*_) typically varies in the range of $1\,K{\Omega }\,-\,100\,K{\Omega }$, depending on the condition of the skin, presence of moisture and other factors^25,26^, whereas the lumped resistance for the layers (*R*_*body*_) is ~100–400 $\Omega$^25,27^.

### Electro-Quasistatic Data Transmission

For both capacitive as well as galvanic coupling, the potential difference created by the magnetic fields is ignored since no closed coupling loops exist at the transmitting or receiving electrodes. Hence, below a certain frequency (*f*) limit, magnetic fields would not contribute to the data transfer allowing electro-quasistatic (EQS) transmission through the human body. The ratio between the magnitudes of the developed electric field ($\overrightarrow{E}$) and the approximation error (${\overrightarrow{E}}_{error}$) in the case of EQS transmission is given as^28,29^:

1 $$\overrightarrow{E}={\overrightarrow{E}}_{EQS}+{\overrightarrow{E}}_{error},\,\frac{{E}_{error}}{E}={\omega }^{2}\mu \epsilon {r}_{tx}^{2}$$

In Eq. 1, *r*_*tx*_ represents the dimension of the transmit device for EQS-HBC (*r*_*tx*_ ~ 0.02 m, refer to Methods Section), $\epsilon$ and *μ* denotes the permittivity and permeability, respectively, of the medium (conductive tissue layer of the human body, in this case). The maximum relative permittivity (for the worst case) of the tissue layers of the human body is ~3000. The nearfield quasi-static approximation ($\overrightarrow{E}\approx {\overrightarrow{E}}_{EQS}$) holds good as long as the magnitude of ${E}_{error}\ll E$, which implies:

2 $${\omega }^{2}{\mu }_{tissue}{\epsilon }_{tissue}{r}_{tx}^{2}\ll 1,\,{\epsilon }_{tissue}\approx 3000{\epsilon }_{air},\,{\mu }_{tissue}\approx {\mu }_{air}$$

3 $${f}_{EQS-HBC}\ll \frac{1}{2\pi {r}_{tx}\sqrt{{\mu }_{tissue}{\epsilon }_{tissue}}}\approx 43.61\,MHz.$$

As seen from Eqns 2 and 3 ^30,31^, considering $c=3\,\ast \,{10}^{8}\,m/s$ as the velocity of propagation of EM waves in air, the intensity of the electromagnetic fields radiated is dominated by the quasi-static nearfield, as long as ${f}_{EQS-HBC}\ll 43.61\,MHz$. It should, however, be noted that biological tissue is dispersive, and the threshold frequency could vary. In this work, we employ transmission frequency of 1 *MHz* thereby allowing quasi-static field $({\rm{approximation}}\,{\rm{error}}={(\frac{1MHz}{43.61MHz})}^{2}\,\ast \,100=0.05 \% )$ as the dominant mode of propagation through the body and thus enabling electro-quasistatic human body communication (EQS-HBC).

On the other hand, for the EQS-HBC leakage, considering the human body as an antenna (maximum height of the human body ${r}_{body} < 2\,m$), and that the leakage signal out of the human body is being picked up in the air medium (${\epsilon }_{air}=1$), the threshold frequency evaluates to ${f}_{QSL}\ll 23.88\,MHz$. This yields an approximation error of ~0.2% for the quasi-static assumption of the leakage signal at 1 MHz.

### Steganographic Covert Communication

Steganography is a form of covert communication which hides the transmitted data from a third party even without encryption. In the context of wireless communications, spread spectrum techniques to hide information in channel noise have been explored which comes at the expense of extra communication energy^32^. Analogously, while the EQS-HBC transmitted signals suffer low loss, the leaked EQS-HBC signals are concealed within noise for an attacker, thereby showing promise to enable covert steganographic communication in the form of an inherent physical layer security.

## EM Radiation in WBAN and Side-channel Quasi-Static Leakage in EQS-HBC

In traditional WBANs, the transmitter radios are designed to transmit data wirelessly as far as possible over air, instead of restricting the transmission to the body, which makes it inherently insecure. Even in presence of encryption, there have been several vulnerabilities of different radio protocols for wearable and implantable devices^33^.

In the case of EQS-HBC, although the transmitted broadband signal suffers loss due to the weak capacitive return path between the transmitter and the receiver^34^, the loss is significantly lower than wireless signal propagation. Hence the received signal can be reliably decoded by an interference-robust receiver^35,36^. From the security perspective of EQS-HBC, if it can confine the data transmission within the human body, it would enable a form of physical layer security, which is presently non-existent in WBANs. Thus, the data transmission would be fully secure from an external malicious attacker. The adversary needs to be in direct physical contact with or almost touching the person to gather any EQS-HBC data. This will enable secure and covert communication without any overhead, with orders of magnitude lower energy than WBAN, which is currently non-existent. EQS-HBC introduces a basis for physical security. An additional layer of mathematical security (i.e. encryption) may or may not be added depending on the application scenario and trust factors. For instance, if it can be ensured that an adversary cannot touch the human without his/her knowledge during EQS-HBC, no additional encryption will be necessary.

To evaluate the inherent data security and privacy of the EQS-HBC transceiver system, we need to analyze whether any signal in the form of quasi-static “side-channel” is being leaked from the human body during EQS-HBC. Figure 1 provides an overview of the private space for EQS-HBC and WBAN in the presence of an external adversary who can detect the leaked (‘radiated’ in the case of WBAN) QS signals and can attempt to obtain the critical information.

A series of experiments in time and frequency domains are performed to determine this critical leakage during EQS-HBC. The methods are elaborated along with the experiments and results.

## Results

The main goal of performing the experiments is to analyze if the body itself leaks information during the electro-quasistatic human body communication (EQS-HBC). As discussed earlier, capacitive EQS-HBC shows lower channel loss than the galvanic HBC over long distances in the body, and hence our experiments are with capacitive EQS-HBC. The EQS-HBC transmit device (Fig. 1) is built using off-the-shelf components, and consists of a communication module, processing module, memory, power source, and an interface with the human body. The details of the set-up and the EQS-HBC transmit device are discussed in the Methods section. An interfacing band consisting of copper electrode couples the transmitted signals into the body. The received EQS-HBC signal and the QS leakage (QSL) is then measured from other parts of the body using voltage probes or antenna as appropriate, and the probing positions are specified for individual experiments. It should be noted that in a few experiments, QS leakage is reported with direct probe contact (*d* = 0), which is to demonstrate the amount of leakage at the source of the leakage signal.

### Time-domain correlational analysis of QS Leakage Signature

In this experiment, the goal is to examine if any QS leakage can be detected during EQS-HBC data transmission.

**Figure 2:**
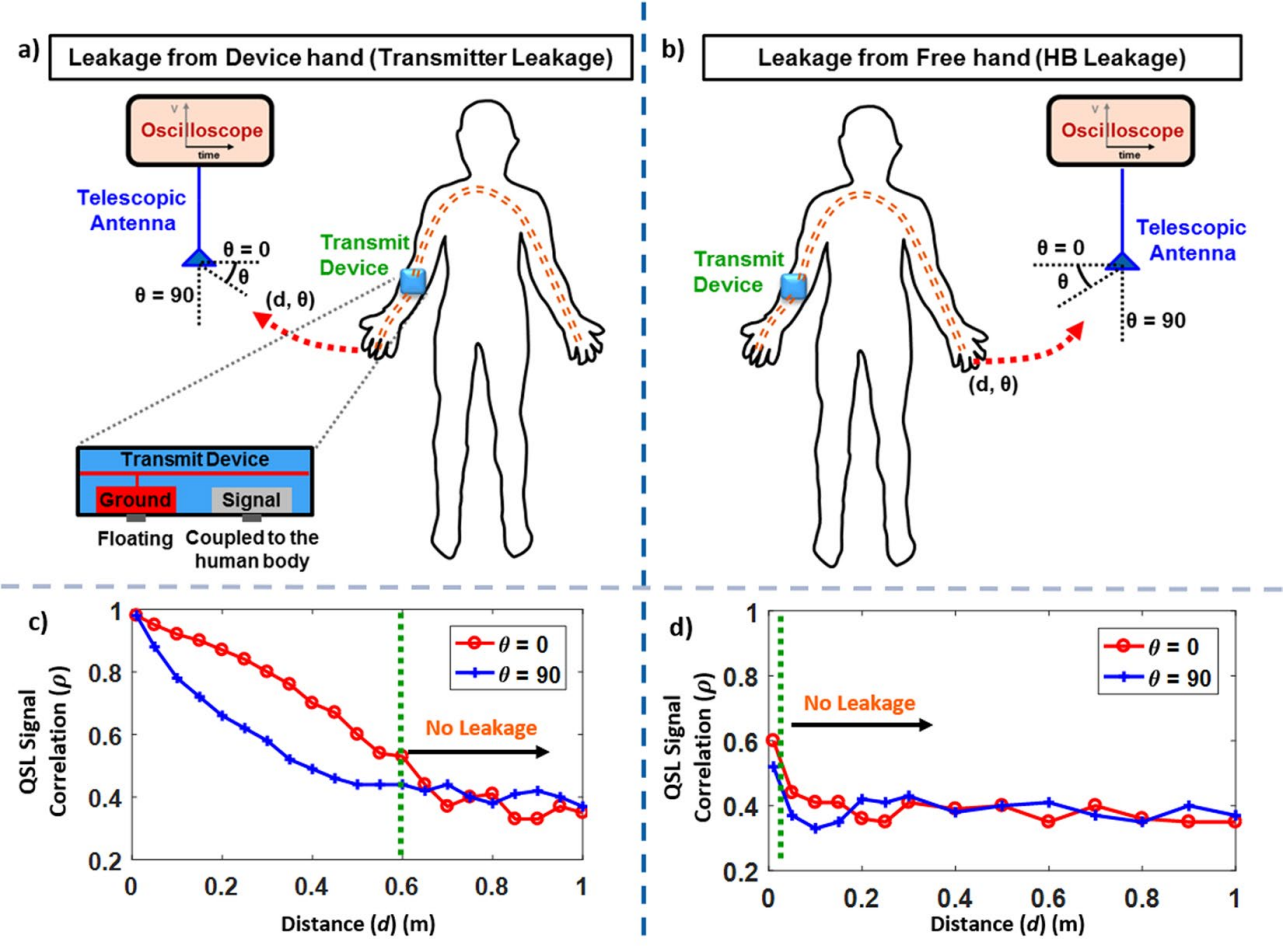
(**a**–**d**) Time-domain Measurements of capacitive EQS-HBC Quasi-static Leakage (QSL) using Oscilloscope with the transmitter wearable device on the device arm. (**a**,**b**) Simplified experimental set-ups to measure the QS leakage from the device and free hands respectively. (**c**,**d**) Voltage Correlational analysis of the measured QS leakage for the device and free hands respectively, with varying angles (*θ*) and distances (*d*) between the antenna and the hands. The measured QS leakage from the device hand is dominated by the leakage due to the EQS-HBC transmitter, while the free hand leakage corresponds to QS leakage due to the human body (HB) alone.

As shown in Fig. 2, the EQS-HBC transmit electrode is coupled to the human forearm (device arm). The transmitter (microcontroller) is excited with a pseudorandom binary sequence (PRBS) at 1 MHz, and using an oscilloscope and a telescopic antenna, the auto-correlation ($\rho$) between the known PRBS data sequence and the QSL signal is measured with varying distances (*d*) away from the body and two angles ($\theta =0^\circ$: parallel to the antenna, $\,\theta =90^\circ$: perpendicular to the antenna) between the device hand and the antenna, as shown in Fig. 2(a). Next, the QS leakage from the free hand is measured with varying distances between the free hand and the antenna connected to the oscilloscope (Fig. 2(b)).

**Figure 3:**
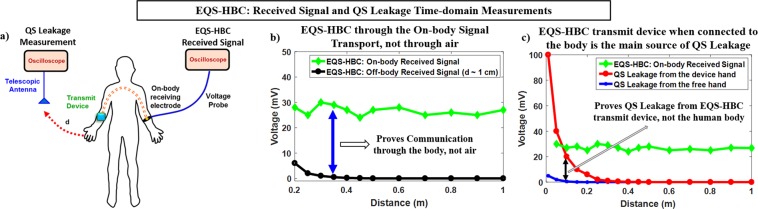
(**a**–**c**) EQS-HBC Signal Transmission (*V*_*EQS-HBC*_) and Quasi-static Leakage (*V*_*QSL*_) Signal Measurement with distance in time-domain using an oscilloscope, voltage probe, and an antenna. The transmission signal amplitude is 3.3 V. (**a**) EQS-HBC Received signal at different on-body locations is ~30 mV (green curve) showing a channel loss of ~40 dB which is almost independent of the distance between the transmitter and receiver. Off-body signal corresponding to each of the human body receiver locations is measured in air with very close proximity from the body (*d*_*off*–*body*_ ~ 0.01 m) (black curve). This shows that the EQS-HBC occurs through the on-body signal transmission, and not through the air. (**b**) The EQS-HBC signal received at different locations of the body is ~30 mV (green curve). Quasi-static Leakage around the body is measured in air medium from both device hand (red curve) and free hand (blue curve) respectively. The QS leakage (QSL) measurement set-up is shown in Fig. 10. Note that for the EQS-HBC received signal measurement, distance refers to the on-body distance between the transmit device and the receiving electrode. In the case of leakage measurements here, it is the distance between the antenna and the corresponding hand for which the leakage is measured. The free hand, although contains almost the same amount of signal, leaks considerably lesser than the device hand, proving that human body alone does not leak. However, the human body aids the transmit device to leak (device hand leakage) by providing a low impedance closed path with the earth ground, which will be discussed in the next experiments.

Correlational analyses for the QS signals leaked during EQS-HBC from the device hand and the free hand respectively, are shown in Fig. 2(c,d). From Fig. 2(c), it can be seen that while the QS leakage from the hand with unshielded EQS-HBC device is detectable up to ~0.5 m, EQS-HBC signals contained in the free hand does not leak beyond ~0.01 m, although both the hands contain the same amount of EQS-HBC signal (Fig. 3 – green curve).

The above observations *prove that the human body itself does not leak*, *but the EQS*-*HBC transmitter is the source of the QS leakage*. However, this experiment does not provide conclusive proof if the transmitter itself leaks the signals.

### Time-domain Measurements of EQS-HBC Received Signal and the QS Leakage Signature

In this experiment, the goal is to check whether both the device hand and the free hand contains the same amount of signal, and also that the EQS-HBC signals are transmitted through the human body and not through the air.

The received EQS-HBC signal in different locations (varying distances) on the body is measured, as shown in Fig. 3(a) (green curve). Corresponding to each on-body location, the off-body quasi-static (QS) leakage is also measured in very close proximity (*d*_*off*–*body*_ ~ 0.01 m) to that location. Figure 3(a) shows the amount of received signal for EQS-HBC is almost independent of the distance between the transmitter and the receiver, which is expected in capacitive human body communication^37^. The off-body signal measured in air at very close proximity corresponding to each of the receiving locations is very small compared to the on-body received signal. This clearly shows that the EQS-HBC communication is established through the body and not through the air.

This experiment (Fig. 3(b)) shows that the QS leakage from the device hand (red curve) is considerably higher than the free hand (blue curve), which is consistent with the correlational analysis from the previous experiment (Fig. 2). This is a very fascinating observation since both the hands contain almost the same amount of EQS-HBC signal (Fig. 3(a) - green curve).

Another important point to note from Fig. 3(b) is that at very small distances, although the leakage measured is high, the on-body signal measured remains same. This is because of the weak capacitive return path in case of EQS-HBC communication.

### Shielded Standalone Transmitter QS Leakage

From the previous experiments, it is evident that the EQS-HBC transmitter leaks. This goal of this experiment is to investigate whether the standalone transmitter leaks by itself, that is, without the human body connected to it.

To perform this measurement, the effect of the connected wires needs to be eliminated. Hence, the transmitter is shielded using a copper-coated box forming a Faraday cage, with the shield (Sh) (refer to the circuit modeling sub-section) connected to a fixed potential. The shield thus hides the ground plane (N) of the EQS-HBC transmitter from coupling directly to the Earth ground (low coupling capacitance *C*_*gn*_), thereby ensuring that the effect of QS fields emanating from the transmit device and the connected wires are eliminated. However, since the shield needs to be connected to a fixed potential, the transmitter ground (N) is connected to the shield, which now forms a capacitive path (*C*_*gsh*_) with the earth ground. Without any contact with the human body, the standalone transmitter device is powered on with a 1 *MHz* PRBS signal, and the leakage from the device is measured using a spectrum analyzer (SA) and an antenna connected through a wideband amplifier. It should be noted that SA provides a low impedance termination (50 *Ω*) and hence both the EQS-HBC received power and the quasi-static (QS) leakage power measured is less. However, it provides a fair comparison for different measurement modalities and to root-cause the source of the QS leakage during EQS-HBC. We can expect that if the shielded transmitter without EQS-HBC shows high QS leakage, it can be concluded that the standalone transmitter itself leaks.

**Figure 4:**
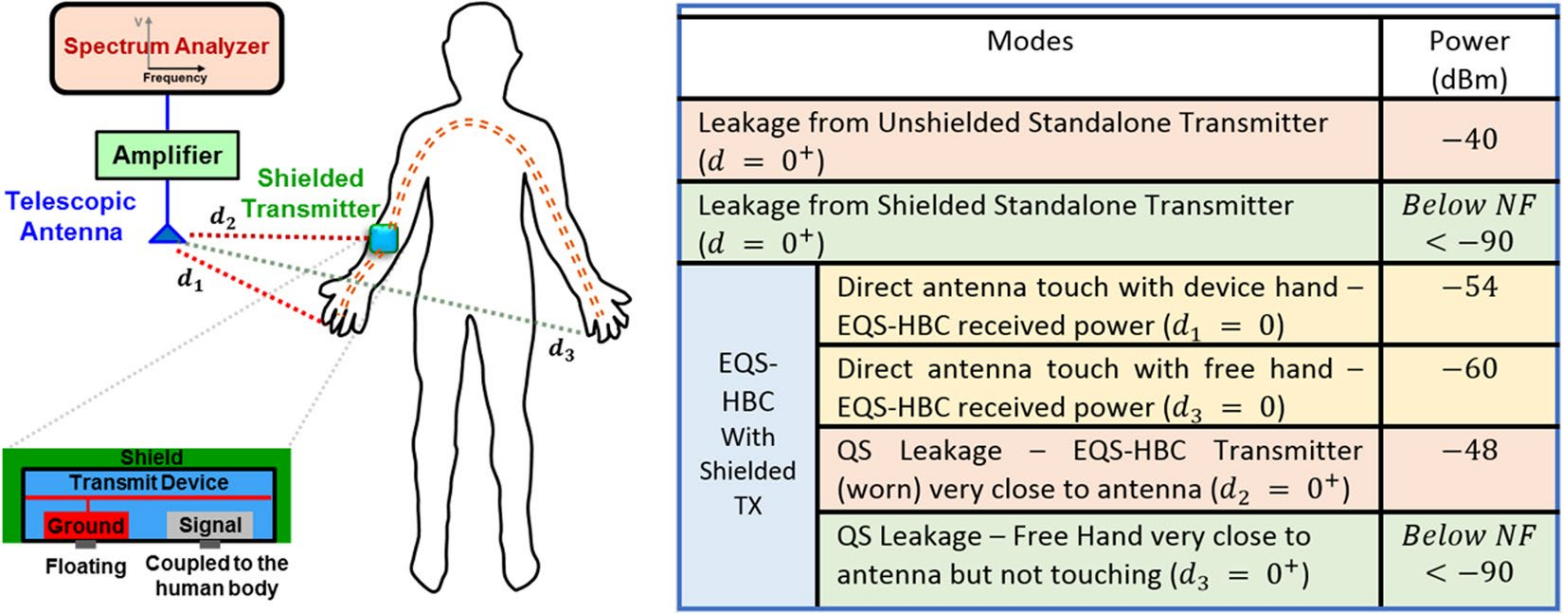
Spectrum Analyzer Measurement shows that the shielded standalone transmitter does not radiate. However, the transmitter when worn on the human body for EQS-HBC shows significant leakage (−48 *dBm*). Note that for the capacitive EQS-HBC, although both the device and free hands contain similar amount of signal (−54 *dBm*, −60 *dBm* respectively), the free hand shows negligible QS leakage (<−90 *dBm*).

As seen from the table in Fig. 4, the unshielded standalone transmitter leaks significantly (−$40\,dBm$), when in close proximity to the antenna ($d={0}^{+}$), whereas the shielded transmitter shows negligible signal leakage (below noise floor: <−$90\,dBm$ at ${d}_{3}={0}^{+}$).

*The above observations confirm that the shielded standalone transmitter does not leak any* QS *signal*.

### Shielded Transmitter Leakage during EQS-HBC

The goal of this experiment is to examine whether the shielded transmitter in contact with the human body causes the quasi-static leakage during EQS-HBC data transmission.

The shielded transmitter is worn on the forearm (device arm) for EQS-HBC. Using the same set-up as in experiment 3 (Fig. 4), with the SA and the antenna, the signal level for the case of direct contact with the device hand (*d*_1_ = 0) and the free hand (*d*_3_ = 0) is measured respectively. It can be seen from Fig. 4 that the signal power contained as measured in the SA is −$54\,dBm$ in the device hand and −$60\,dBm$ in the free hand. As expected, the path loss is lower for capacitive mode, and both the hands contain similar amount of the EQS-HBC signals. As the EQS-HBC transmitter on the device hand is brought in close proximity to the antenna (*d*_2_ = 0^+^), the QS leakage is significant (−$40\,dBm$), as seen from Fig. 4. Another fascinating observation is the fact that the signal contained in the free hand (−$60\,dBm$) immediately dies down (below noise floor of the SA) within a few mm, as the leakage signal power measured from the free hand at close proximity of the antenna (*d*_3_ = 0^+^) is negligible (below noise floor: <−$90\,dBm$).

The above observations from this experiment confirm that even after shielding, the transmit device when connected to the human body causes the QS leakage.

From the above experiments, it is clear that *neither the human body alone*, *nor the transmitter itself leaks the signals*. However, the *EQS*-*HBC transmitter* (*even after shielding*) *in contact with the human body shows leakage*.

### EQS-HBC Quasi-Static Leakage Field Distribution

**Figure 5:**
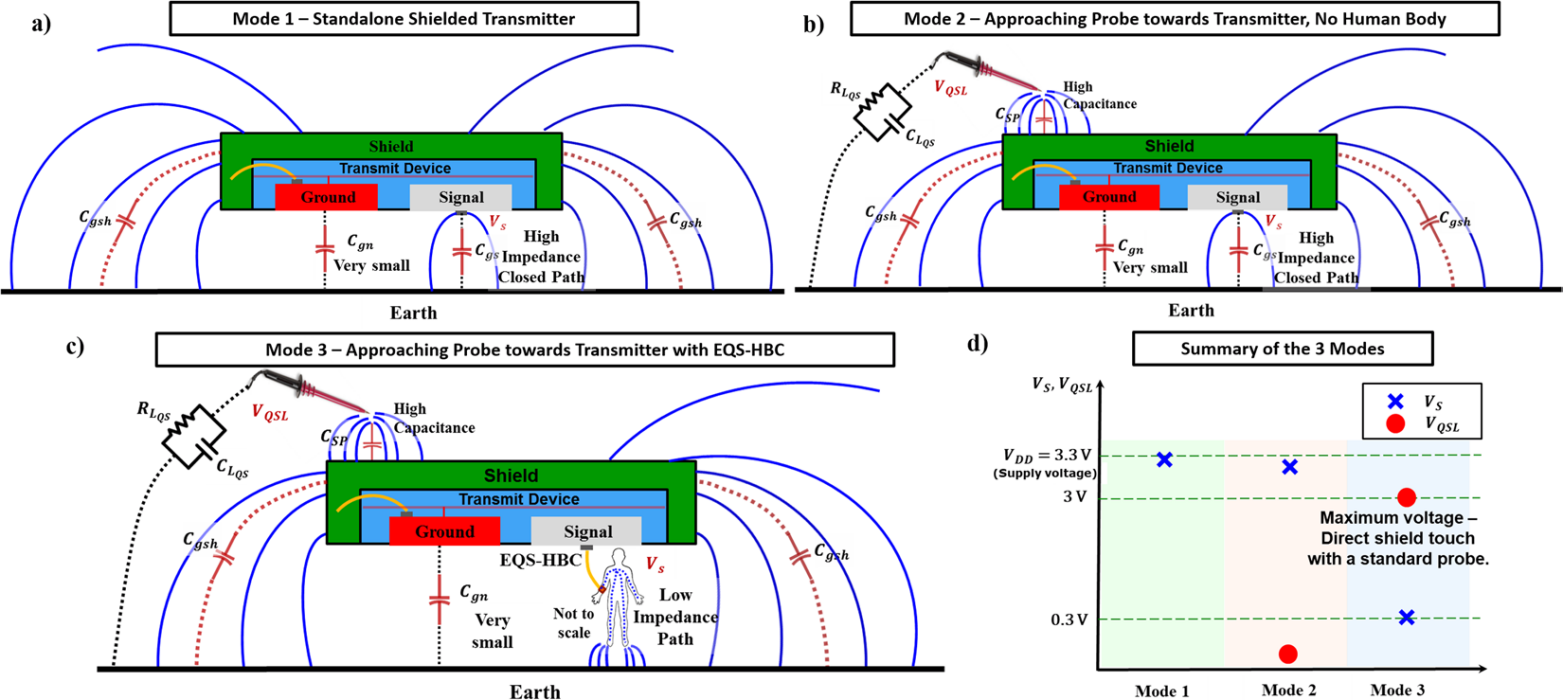
(**a**–**d**) QS Field distributions for different configurations of the transmitter device. (**a**) In mode 1, voltage drop across the signal plate of the Standalone shielded transmitter and earth ground is maximum (*V*_*S*_ ~ *V*_*DD*_) as there is no direct path from the signal plate to the earth ground. (**b**) In mode 2, as an attacker approaches with a probe towards the shielded transmitter, it receives negligible voltage as no current flows due to the high impedance path from signal to ground. Hence, standalone shielded transmitter does not leak. (**c**) In mode 3, Human body coupled to the transmitter device for EQS-HBC provides a low resistance closed path to ground; hence higher voltage received by the attacker (*V*_*QSL*_) (**d**) Summary of the 3 modes – In absence of the human body, all the voltage drop (*V*_*S*_) occurs across the signal terminal and ground and the attacker does not pick any signal (mode 2). In presence of the human body, a close-by attacker (touching the shield) can obtain a high signal. Hence, in spite of shielding, the EQS-HBC transmitter device leaks.

The goal of the field distribution analysis (Fig. 5) is to explain the basis behind the observations in previous experiments for different configurations of the transmit device.

Figure 5(a,b) shows the electric field distributions due to the standalone transmitter device without any body contact. For mode 1, in absence of the measuring probe (no nearby attacker) and without the human body, the closed path from the small ‘Signal’ plate of the transmitter to the Earth ground is formed by a very weak coupling capacitance (*C*_*gs*_). Hence the fields formed between the signal terminal and the Earth ground are very weak, and significant portion of the transmitted signal voltage drop occurs across the Signal plate and the Earth ground (${V}_{S}\simeq {V}_{DD}$, Supply Voltage). As an attacker approaches to intercept the data being transmitted, the probe forms a low impedance path between the shield (connected to the ground terminal of the transmitter) and the Earth ground. However, the signal plate to the Earth ground still presents a high impedance path, and most of the signal still drops across *C*_*gs*_, i.e. ${V}_{S}\le {V}_{DD}$. Hence the attacker can only receive negligible amount of signal $\,{V}_{QSL}\simeq 0$.

Figure 5(c) analyzes the field distributions due to the transmitter device during EQS-HBC data transmission in presence of the probe (emulating an attacker). In mode 3, the human body forms a low resistance path between the small Signal plate to the Earth ground which now allows current to flow. In this case, the attacker obtains the maximum amount of the transmitted signal ${V}_{QSL} \sim 3V$, using voltage probes and an oscilloscope with high termination impedance ($10\,M\Omega$).

*The above analyses infer that the human body alone does not leak*, *and the shielded standalone transmitter device does not radiate*. *However interestingly*, *even the shielded transmitter leaks during EQS*-*HBC*, *when in contact with the human body*, *which is explained through the QS field theoretic viewpoint* (Fig. 5(c)). *This observation conclusively suggests that the human body is aiding the transmitter to leak information*.

### Theoretic Circuit Modelling of the EQS-HBC Leakage and Experimental Validation

**Figure 6:**
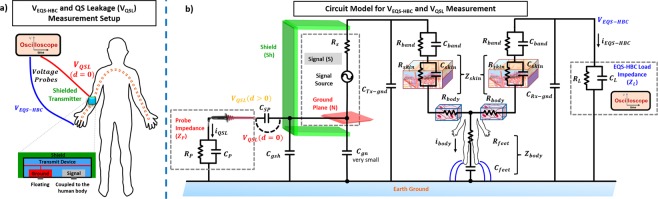
(**a**) EQS-HBC Measurement set-up with the shielded transmitter in the wrist (device arm) and (**b**) its corresponding circuit model. The impedances for the skin and tissue layers^26^ are modelled, along with the signal sources, copper electrode coupler (band) and the measurement probes, to form the complete circuit model for EQS-HBC. Note that the probe is directly connected (*d* = 0) to the human body to measure the signal level from the source of the leakage. The EQS-HBC received voltage is measured from the fingers of the device hand.

The goal now is to develop a circuit model for EQS-HBC (Fig. 6(b)) to further analyze the cause of the QS leakage and to implement countermeasures for reducing the “side-channel” leakage information.

In the case of EQS-HBC, the signal transmission is dominantly electro-quasistatic and hence the lumped circuit approximation holds (since wavelength $\lambda  \sim \frac{3\,\ast \,{10}^{8}\,m/s}{\sqrt{8}\,\ast \,1\,MHz} \sim 105\,m$ of the transmitted signal is much greater than the length (*l* ~ 2 *m*) of the transmission channel, that is, human body). The different elements of the circuit model (Fig. 6(b)) include the signal generator source resistance (*R*_*s*_), the band to skin capacitance (*C*_*band*_), skin layer resistance (*R*_*skin*_), skin layer capacitance (*C*_*skin*_), body resistance (*R*_*body*_), body to feet resistance (*R*_*feet*_), feet to ground capacitance (*C*_*feet*_), body to ground capacitance (*C*_*Tx-gnd*_, *C*_*Rx-gnd*_), shield (connected to transmitter ground) to earth return path capacitance (*C*_*gsh*_) and the load impedance due to the probes (*Z*_*P*_, *Z*_*L*_) for measuring the leakage and the EQS-HBC received voltage respectively. The source impedance of the signal generator ${R}_{s}=50\,\Omega$. The band capacitance is formed due to the small air gap (d) between the transmitter electrode and the skin, which is in ~200 pF considering the electrode size of ~0.0004 m^2^ and $d=0.01\,mm$. The skin layer resistance ${R}_{skin}\sim 10\,K\Omega$ and typically varies in the range of $1\,K\Omega \,-\,100\,K\Omega$^25,26^, depending on the skin moisture and other factors. The skin layer thickness is in the range of 0.1–4 *mm*, and considering skin area of ~0.0004 m^2^ near the EQS-HBC transmit device, the skin layer capacitance (*C*_*skin*_) can be computed to be in the range of ~100 *pF*-1 *nF*^38^. The body resistance (*R*_*body*_)) is in the range of 100–400 $\Omega$^25,27^. The resistance of the tissue could depend on the on-body transmission distance. However, it does not affect the EQS-HBC channel loss or the measured QS leakage. The feet to ground capacitance *C*_*feet*_ ~ 10 - 20 *pF*, considering a feet area of ~0.01 m^2^ and a feet to ground separation of 0.01 m^38^. The measurement probes are modelled as the load (*Z*_*P*_, *Z*_*L*_ respectively) for both the QS leakage and EQS-HBC received voltage signal. In this set of experiments, the shield (Sh) is connected to the ground (N) of the transmitter device.

The QS leakage and the received EQS-HBC voltage signal are measured using an oscilloscope by putting a voltage probe on the shield (direct contact) and the device hand respectively (Fig. 6(a)). Although the shielding significantly reduces the return path capacitance between the transmitter ground and the earth ground (*C*_*g*_), the shield capacitively couples to the earth’s ground through *C*_*gsh*_. Hence, shielding the EQS-HBC transmitter does not eliminate the unnecessary QS leakage, which has been demonstrated in previous experiments.

**Figure 7:**
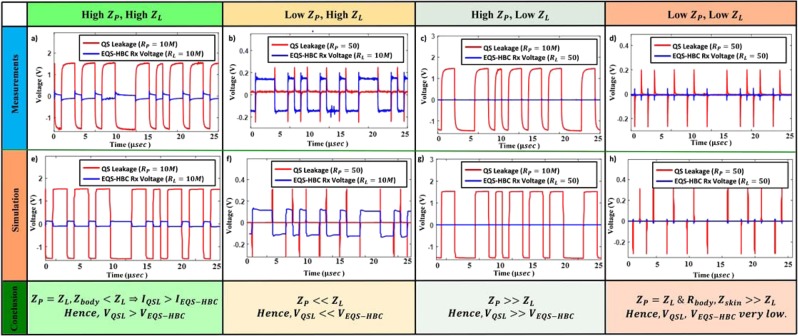
(**a**–**d**) Measured oscilloscope signals with the EQS-HBC set-up shown in Fig. 6(a), for different termination for both the QS leakage and the EQS-HBC received voltage. (**e**–**h**) Proposed Circuit Model (Fig. 6(b)) simulation waveforms for the same set of loading constraints. The simulation results complement the actual measurements for all different conditions, proving that the model is accurate. Note that the QS signature is inverted to the actual transmitted signal.

Figure 7(a–d) shows the oscilloscope captured waveforms with the shielded transmitter prototype, for 4 different load combinations (2 probes each having 2 impedances: $10\,M\Omega$ and $50\,\Omega$), for both the QS leakage and the EQS-HBC received voltage. Note that both probes are connected for measuring the QS leakage and the received HBC voltage simultaneously. In Fig. 7(a), ${R}_{P}={R}_{L}=10\,M\Omega$, and, ${Z}_{body} < {Z}_{L}$; hence the current and voltage received by the probe is higher than the EQS-HBC current ${I}_{QSL} > {I}_{EQS-HBC}$, and $\,{V}_{QSL} > {V}_{EQS-HBC}$. When the probe impedance (${R}_{P}=50\,\Omega$) is significantly lower than the load impedance for EQS-HBC (${R}_{L}=10\,M\Omega$), ${Z}_{P}\ll {Z}_{L}$, hence $\,{V}_{QSL}\ll {V}_{EQS-HBC}$ (Fig. 7(b)). Similarly, when the receiver EQS-HBC load impedance (${R}_{L}=50\,\Omega$) is much lower than the probe impedance (${R}_{P}=10\,M\Omega$), ${Z}_{P}\gg {Z}_{L}$, and hence $\,{V}_{QSL}\gg {V}_{EQS-HBC}$ (Fig. 7(c)). Finally, as seen from (Fig. 7(d)), when both ${Z}_{P}$ and ${Z}_{L}$ are low (${Z}_{P}\gg {Z}_{L}=50\,\Omega$), ${R}_{body}\gg {Z}_{L}$ and ${Z}_{skin}\gg {Z}_{L}$, and hence the maximum drop occurs across the skin and body impedances. So, both ${V}_{EM}$ and ${V}_{EQS-HBC}$ are very small, although $\,{V}_{QSL}\gtrsim {V}_{EQS-HBC}$, since ${I}_{QSL} \sim {I}_{EQS-HBC}+{I}_{body}$.

The simulation results of our proposed EQS-HBC circuit model (Fig. 7(e–h)) closely matches the actual measurement results in terms of the voltage swing for both the received EQS-HBC signal and the EQS-HBC QS leakage with varying load conditions. This not only shows that the proposed EQS-HBC circuit model is accurate, but also confirms that the QS leakage signature is inverted. The inversion of QS leakage signature is due to the fact that the probe directly couples with the ground terminal (N) of the transmitter device, which is picked up by an attacker.

### Countermeasure against the EQS-HBC transmitter QS Leakage & Experimental Validation

The goal is to develop a countermeasure against the EQS-HBC transmitter leakage. From the experiments and the developed circuit theoretic models, it is confirmed that the EQS-HBC transmitter leaks only when aided by the human body. Now, we demonstrate a technique to reduce this QS leakage.

**Figure 8:**
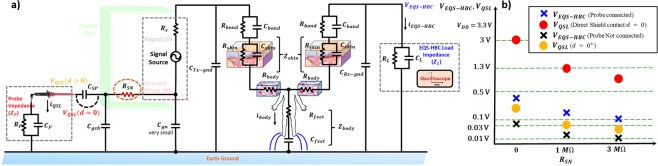
(**a**,**b**) Countermeasure against EQS-HBC leakage. (**a**) A high resistance (*R*_*SN*_) de-couples the transmitter ground plane and the shield. (**b**) EM (*V*_*QSL*_) and EQS-HBC voltage (*V*_*EQS*–*HBC*_) levels are measured against different values of *R*_*SN*_. As *R*_*SN*_ is increased, both *V*_*QSL*_ and *V*_*EQS*–*HBC*_ reduces. Beyond a certain value of *R*_*SN*_, the EQS-HBC received signal gets reduced and can no longer be decoded. Hence, there exists an optimum between the area of the shield connected with transmitter ground through *R*_*SN*_, and the remaining area that connects to the transmitter ground directly, so as to minimize the EM leakage while maintaining reliable EQS-HBC.

As shown in Fig. 8(a), a high resistance (${R}_{SN}$) is inserted in series to de-couple the shield (Sh) from the ground terminal (N) of the transmitter. As most of the voltage signal is dropped across $\,{R}_{SN}$, ${V}_{QSL}$ reduces significantly, as seen from Fig. 8(b). However, the EQS-HBC received voltage (${V}_{EQS-HBC}$) also reduces as the current in the return path gets reduced.

When the resistance ${R}_{SN}=0$ (without the countermeasure - Fig. 6(a)), with both QS and EQS-HBC probes connected at the shield and the fingers of the device hand respectively, the received EQS-HBC voltage (${V}_{EQS-HBC}$) is $300\,mV$, while the signal from the source of the QS leakage (probe directly connected to the transmitter shield: $\,d\,=\,0$) is ${V}_{QSL} \sim 3\,V$ (Fig. 8(b)). With the probe disconnected, the EQS-HBC received signal level is ~$30\,mV$, which can be reliably decoded in the EQS-HBC receiver device. When the probe is not in direct contact to the transmitter shield but is in close proximity ($d\,=\,{0}^{+}$), the amount of QS leakage signal received is ~$170\,mV$. As the series resistance (${R}_{SN}$) is inserted and increased, both ${V}_{QSL}$ and ${V}_{EQS-HBC}$ gets reduced, and beyond ${R}_{SN}=3\,M\Omega$, ${V}_{EQS-HBC}$ goes below 10 mV, and the accurate detection becomes significantly harder for the EQS-HBC receiver.

Hence, connecting the entire shield with a high resistance to ground is not a judicious solution as it can impede EQS-HBC. Also, having the shield fully connected to the ground potential of the transmitter leaks QS signals, which may be intercepted by an almost-touching adversary.

The above observations infer that there exists an optimization between the size of the shield plane that can be directly connected to the transmitter ground, and rest of the shield plane connected to the ground plane through the high resistance *R*_*SN*_. Depending on the application and device form factor, the optimum shield sizes, pattern and ground plane size can be customized based on the fundamental understanding and models developed in this work.

### Privacy Space Comparison: EQS-HBC vs. WBAN

**Figure 9:**
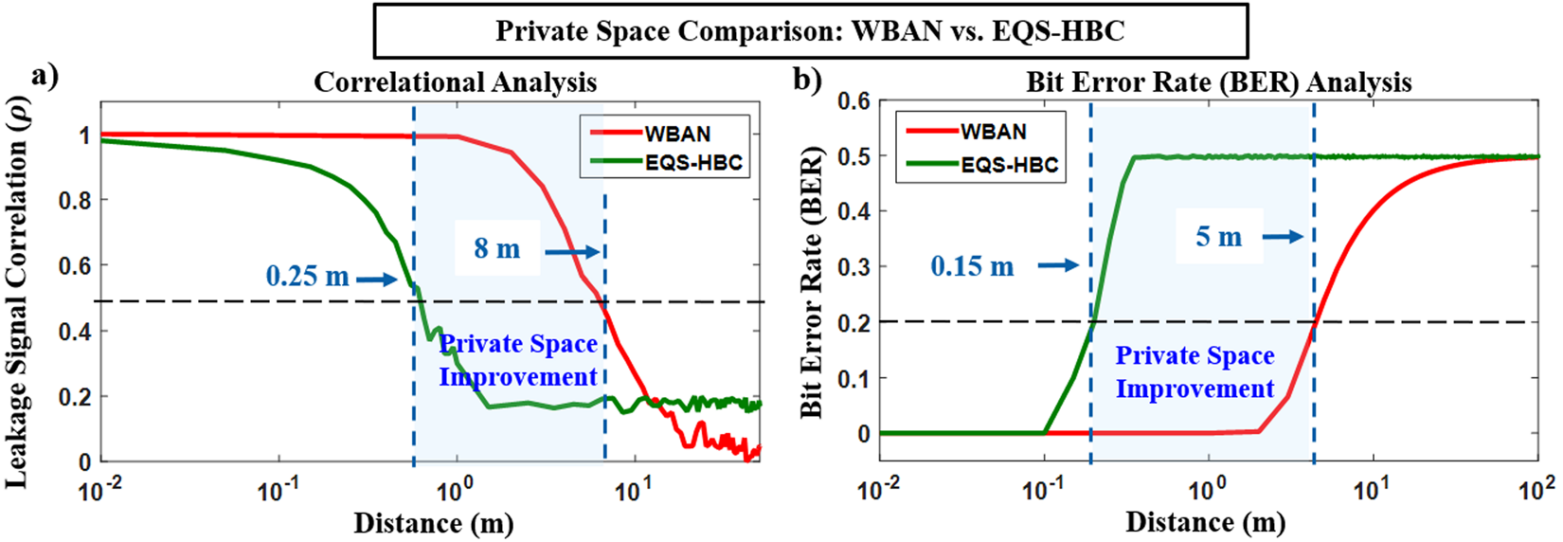
Private Space Comparison for EQS-HBC vs. WBAN. Correlational and BER analysis of the leaked “side-channel” EM signals to determine the range till which an attacker can intercept the transmitted data. EQS-HBC provides >30× improvement in private space over traditional WBANs. The distance is defined from the device hand. Note that the EQS-HBC transmit device signal amplitude is 3.3 V, while the WBAN signal transmission power is −40 dBm. For WBAN, a 2.4 GHz carrier frequency with 1 MHz data rate, and a 6 dB noise figure for the wireless receiver was considered for the analysis. Note that increase in transmit power (>−40 dBm) in the case of WBAN or considering more idealistic loss exponent (*d*^2^) will only increase the range (>5 m) for WBAN signals in which it can be snooped by an attacker, making an even stronger case for EQS-HBC advantage over WBAN in terms of physical security/privacy.

To substantiate the security benefits of EQS-HBC over the traditional WBAN, a private-space comparison is necessary. In a WBAN, signals are *radiated* wirelessly through free space, and even considering a very low transmission power of −$40\,dBm$ and the free space path loss (FSPL) varying with the cube of the transmit distance (${d}^{3}$), a known data sequence can be detected using auto-correlation based techniques over a distance of 8 m, as shown in Fig. 9(a). In the case of EQS-HBC, the QS leakage for a known data sequence can be detected up to a distance of 0.25 m, which is practically very close to physical contact with the person. Although auto-correlation serves for a fair comparison, it is an exaggerated attack model, since it only holds good for a known bit sequence.

Bit Error Rate (BER) analysis (Fig. 9(b)) is a more practical approach that works for any pseudo-random bit sequence (PRBS). For a BER of <0.2 (at most 1 out of 5 bits are incorrect for a long sequence), WBAN signals can be detected up to 5 m in space, whereas EQS-HBC signals can be detected only up to 0.15 m, enabling >30× improvement in private space compared to WBAN. Hence, EQS-HBC provides inherent data privacy and can enable steganographic covert communication.

## Discussion

Different skin conditions/thickness as well as the physiologic states such as highly edematous or dehydrated cachectic conditions can alter the body fluidic conditions. This would change the skin and tissue impedances (${R}_{skin},\,{C}_{skin},\,{R}_{body}$) that are considered in the lumped bio-physical model of EQS-HBC (Fig. 6(b)).

**Figure 10:**
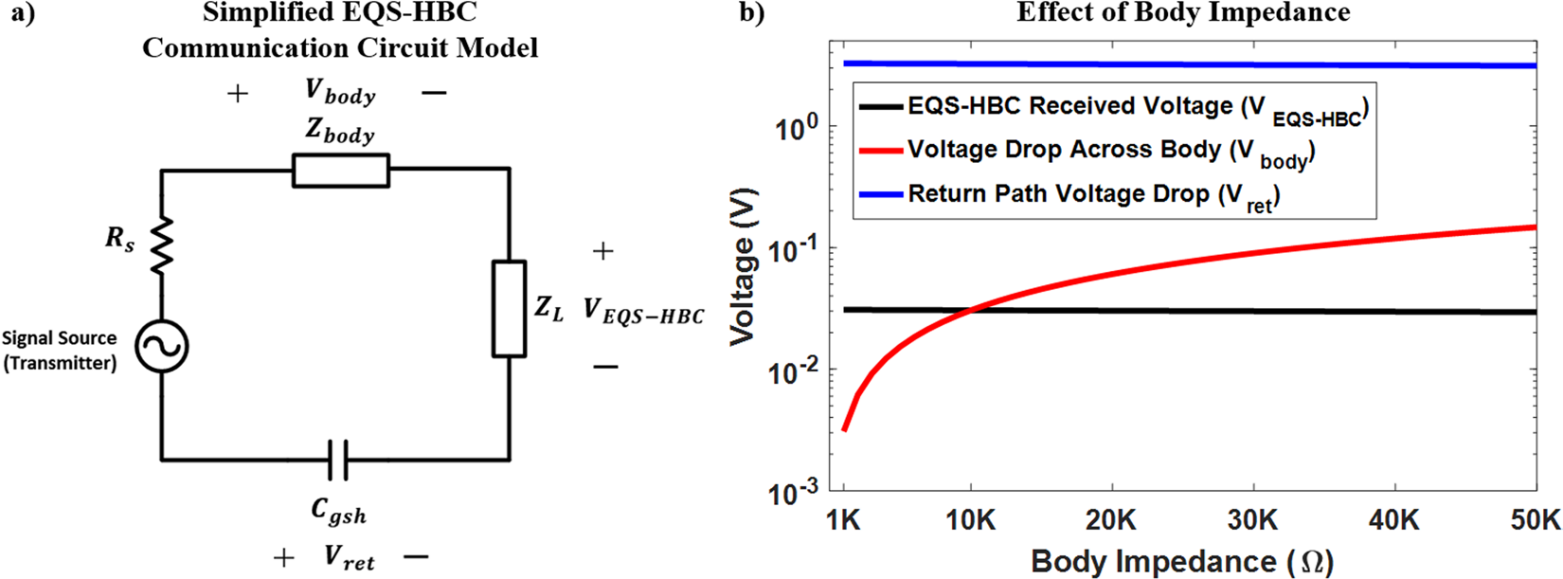
(**a**) Simplified EQS-HBC Circuit model with the forward path components lumped into a single impedance. (**b**) Effect of body impedance on the EQS-HBC received voltage (*V*_*EQS*–*HBC*_), voltage drop across the human body (*V*_*body*_), and the return path voltage drop (across *C*_*gsh*_) for a 3.3V transmitted voltage at 1 MHz. Variation of body impedance in the range of tens of Kiloohms does not affect the EQS-HBC received voltage since the load impedance (*Z*_*L*_) and the return path impedance values are orders of magnitude larger than the forward path body impedance.

The simplified EQS-HBC circuit model with the forward path components lumped into a single impedance is shown in Fig. 10(a). The closed loop in case of capacitively-coupled electro-quasistatic human body communication (EQS-HBC) is formed by the return path capacitance (*C*_*gsh*_) between the transmitter and receiver which is in the order of hundreds of femtofarads (estimation of the return path capacitance (*C*_*gsh*_) is performed by connecting several known value capacitances (*C*_*expt*_) between the identical transmitter and the receiver device ground and measuring the loss for each case). Hence the impedance provided by the return path capacitance is >1 MΩ for the frequency range of <1 MHz. However, all the forward path body components (between the transmitter and receiver) considered in the human body model has impedance values in the order of tens of KΩ^25,26^. Since the return path capacitance has the highest impedance, the closed loop current is primarily determined by its value and is very weakly dependent on the forward path components. Also, the received voltage is measured across the load, which is primarily capacitive (*C*_*L*_) due to the very high resistive component (*R*_*L*_) of the receiver input impedance. So, the measured channel loss is primarily determined by the capacitive division between the load capacitance and the return path capacitance $(\frac{{Z}_{L}}{{Z}_{body}+{Z}_{{C}_{gsh}}+{Z}_{L}}\approx \frac{{C}_{gsh}}{{C}_{gsh}+{C}_{L}},\,{\rm{since}}\,{Z}_{body}\ll {Z}_{{C}_{gsh}}+{Z}_{L})$.

Thus, the communication path loss for EQS-HBC is almost independent of the body impedance, as seen from Fig. 10(b) (black curve – received signal ~30 mV is consistent with the received signal as shown in Fig. 3: green curve). Hence, the received EQS-HBC signal across the body will have the same value irrespective of the body conditions (edematous or cachectic) or different skin thickness, as long as it is terminated by a high impedance load.

## Conclusions

To conclude, this work for the first time, analyzes the security of electro-quasistatic human body communication (EQS-HBC) using the human body as the communication channel. The source of the information leakage is the EQS-HBC transmitter device aided by the human body that provides a low resistance closed path through the earth ground. The Faraday cage, which acts as the shield for the transmitter, reduces the effect of the QS leakage from the standalone transmitter, but it serves as a ground plane to increase both the EQS-HBC received potential as well as the information leakage. A fascinating observation was that although the capacitive EQS-HBC signals from the device hand suffer low loss and maintain similar amplitudes at the receiving end, the QS leakage is negligible even very close to the receiving free hand, proving that the human body does not leak information. The proposed circuit theoretic model for EQS-HBC corroborates all the measurement results and allows for countermeasures to minimize the QS leakage while maximizing the EQS-HBC received potential. De-coupling the shield and the ground of the transmitter using a high resistance reveals that both the QS leakage as well as the EQS-HBC received potential gets reduced. Hence, there exists an optimization between the area of the shield plane that connects directly to the transmitter ground potential, and rest of the shield connected to the ground plane through the high resistance *R*_*SN*_. Finally, we show that WBAN signals can be intercepted even at a distance of 5 m, while EQS-HBC signals can only be detected up to 0.15 m, which is practically in physical contact with the person. Thus, EQS-HBC offers >30× improvement in private space compared to the traditional WBAN, thereby enabling a covert body area network.

## Methods

### Experimental Set-up for the EQS-HBC and QS Leakage Measurement

**Figure 11:**
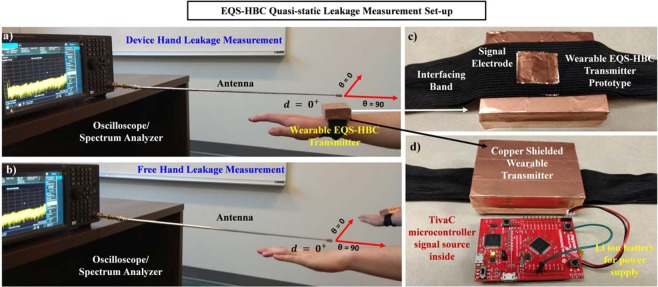
Quasi-static Leakage (QSL) Measurement Set-up with the wearable EQS-HBC device, using an antenna and an oscilloscope. (**a**) Leakage from the device hand is measured with the device arm extended towards the antenna tip and moving away from/towards the antenna. Distance (**d**) is measured between the antenna and the device. This figure shows measurement for *d* = 0^+^ (very close to the antenna, but not touching it). Gradually, the device hand is moved further away from the antenna in two directions (*θ* = 0, 90) and the leakage signal is measured and sent to the PC for further BER/correlational analysis. (**b**) Similarly, leakage from the free hand is measured with distance (**d**) between the free hand tip and the antenna tip. This figure shows measurement for *d* = 0^+^. Note that during the free hand leakage measurement, it is away from the body as well as the device hand to ensure that the leakage from the EQS-HBC device arm do not affect the free arm leakage measurements. (**c**) The shielded wearable EQS-HBC transmit device is shown. It consists of the interfacing band with the copper electrode (signal electrode) which couples the transmitted signal into the body. (**d**) Inside the shield is the ARM Cortex M4 based microcontroller (TivaC) which transmits the data.

The transmitter device for EQS-HBC was developed using off-the-shelf components (Fig. 11(c,d)) and the signal was coupled to the human body (skin) using an interfacing band consisting of copper electrodes (0.02 m × 0.02 m) as discussed earlier in the article. In order to emulate a wearable device, the transmitter is battery-powered. The communication module is implemented using a Texas Instruments LaunchPad evaluation kit (TM4C123G) consisting of an ARM Cortex M4 based microcontroller (TM4C123GH6PM), which transmits the data. A rechargeable Lithium ion battery is used as power supply. The transmitter device was shielded using a copper-coated box to eliminate the QS leakage due to the standalone transmitter. Figure 11(a,b) demonstrates the basic leakage measurement set-up with the wearable EQS-HBC device using an antenna and the oscilloscope (time-domain) or spectrum analyzer (frequency domain). The QS leakage from the device arm is measured with the device arm extended towards the antenna, and the distance (d) is measured between the antenna tip and the body-worn EQS-HBC device. Similarly, for the free hand, leakage is measured with distance as the free hand is extended and moved away/towards the antenna. The extension of the free arm towards the antenna during the leakage measurements ensure that any QS leakage from the device arm do not affect the measurements.

### Safety Limit Compliance in EQS-HBC

**Figure 12:**
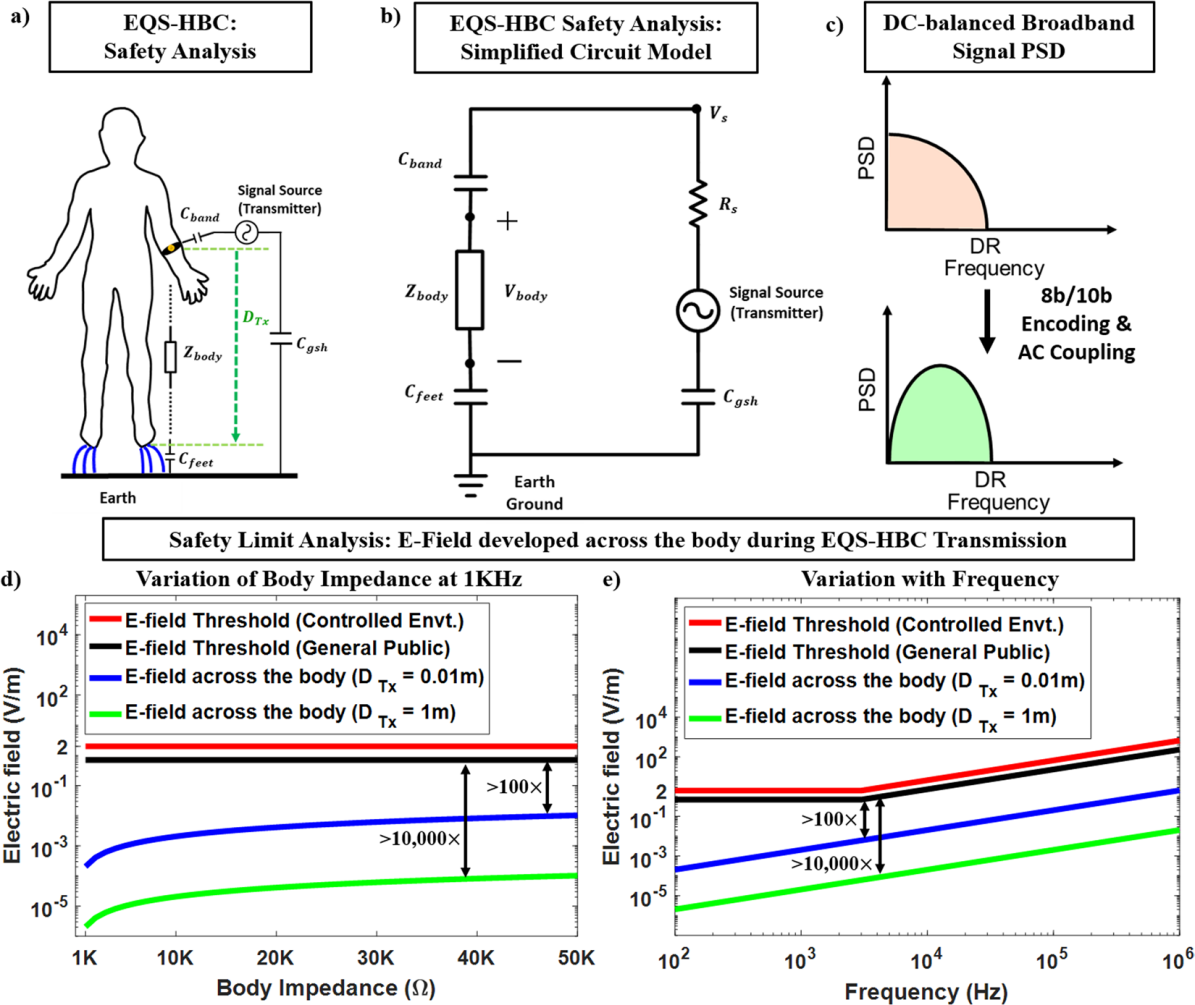
(**a**,**b**) EQS-HBC signal excitation simplified circuit model with the forward path components lumped to a single impedance (*Z*_*body*_). *C*_*band*_ refers to the series coupling capacitor at the output of the transmit device along with the interfacing copper electrode band capacitance formed between the transmit device and the human body, *D*_*Tx*_ denotes the on-body distance for signal transmission from the transmit device to the feet, which would give the voltage drop across the body (*V*_*body*_). (**c**) Power Spectral Density (PSD) of a broadband transmitted signal occupying the complete bandwidth from DC up to the data rate (DR). Bottom: PSD of the broadband transmitted signal after dc-balancing with 8b/10b encoding scheme. (**d**) Electric field developed across the body at a low frequency of 1 KHz is orders of magnitude lower than the IEEE defined threshold of 2.1 V/m (controlled environment) or 0.701 V/m (general public)^39,40^, for varying forward path body impedances (emulating different skin conditions) in the range of few Kiloohms. (**e**) Even with varying frequencies, the developed E-field across the body is orders of magnitude lower than the IEEE defined thresholds^39,40^ at those frequencies.

The circuit model of the EQS-HBC during the signal transmission is shown in Fig. 12(a,b). For safety analysis, only the transmitter is considered as the current distribution is mostly independent of the receiver (high impedance termination). The transmitted signal needs to be biphasic to avoid any harmful electrochemical reactions^39^. For EQS-HBC, the PRBS data sequence is a series of 0’s and 1’s. A series coupling capacitor at the output of the transmit device, along with the interfacing copper electrode band capacitance (*C*_*band*_) formed between the transmit device and the human body provides the AC coupling, as shown in Fig. 12(a). Hence, the signals are converted to AC, and contains both positive and negative phases. The sequence is dc-balanced with the capacitive coupling and 8b/10b encoding scheme, and hence the PSD of the transmitted broadband signal for EQS-HBC (after dc balancing) gets modified as shown in Fig. 12(c). Figure 12(b) shows the simplified circuit model of the EQS-HBC transmission with the forward path components lumped into a single impedance (*Z*_*body*_). *R*_*s*_ denotes the series resistance of the signal source, *C*_*feet*_ represents the capacitance between the feet and the earth’s ground, and *C*_*gsh*_ is the return path capacitance between the shielded transmitter and the earth’s ground. *D*_*Tx*_ is the on-body distance for signal transmission from the transmit device to the feet, which gives the voltage drop across the body (*V*_*body*_).

The safety limit analysis for the developed electric field across the human body is shown in Fig. 12(c,d). The E-field thresholds in compliance with the IEEE standards^39,40^ are also shown in Fig. 12(c,d). Figure 12(c) shows variation of the E-field with the body impedance (emulating the different skin conditions, across different persons, or even time of the day), and Fig. 12(d) shows the E-field for varying frequencies for ${Z}_{body}=10\,K\Omega$ (typical body impedance is in the range of tens of kilo ohms^25,26^). It can be clearly seen that the E-field developed across the body is orders of magnitude lower than the threshold even for ${D}_{Tx}=0.01\,m$ (that is, when the EQS-HBC transmit device is very close to the feet) for different frequencies as well as for varying forward path body impedances.

Also, the amount of current which is perceptible to a human is 1mA ac^25,41^. The ventricular fibrillation threshold is 100 mA and a current of 2 A can cause a cardiac standstill and internal organ damage^25,41^. However, for the case of EQS-HBC, the amount of current passing through the body is in the order of few microamps (μA), since the voltage drop across the body is below 10 mV (as shown in Fig. 10 – red curve) and the body resistance is in the order of tens of kilo ohms. Hence, the current through the body during EQS-HBC is at least three orders of magnitude lower than the perceptible threshold.

The experimental protocols involving human subjects have been approved by the Purdue Institutional Review Board (IRB Protocol #1610018370) and also approved by the Air Force Office of Scientific Research (AFOSR), Department of Defense (DoD) through a 2^nd^ level review. All guidelines and regulations, as given by the Purdue IRB, and AFOSR were followed during the experiments. The authors also confirm that informed consent was obtained from all participants for the experiments.
