# Publisher Correction: Enabling Covert Body Area Network using Electro-Quasistatic Human Body Communication

**DOI:** 10.1038/s41598-020-60493-6

**Published:** 2020-02-27

**Authors:** Debayan Das, Shovan Maity, Baibhab Chatterjee, Shreyas Sen

**Affiliations:** Purdue University, School of Electrical and Computer Engineering, West Lafayette, IN 47907 USA

Correction to: *Scientific Reports* 10.1038/s41598-018-38303-x, published online 11 March 2019

The publication date was omitted from the original PDF version of this Article. This error has now been corrected in the PDF version; the HTML version was correct from the time of publication.

